# Magnetic resonance guided focused ultrasound pallidotomy for Parkinson’s disease

**DOI:** 10.1186/2050-5736-3-S1-O5

**Published:** 2015-06-30

**Authors:** Jin Woo Chang

**Affiliations:** 1 Yonsei University College of Medicine, Seoul, Republic of Korea

## Background/introduction

Parkinson’s disease (PD) is one of the most common movement disorders in adults, often cannot be adequately controlled with medical treatment, and thereby treated with the neurosurgical procedures. Unilateral pallidotomy, making a lesion in the globus pallidus pars interna (GPi), was the predominant surgical technique until early 1900s. Thereafter, deep brain stimulation (DBS) of the subthalamic nucleus (STN) has become a mainstream surgical technique for managing not only the cardinal dopaminergic features of PD, but also levodopa-induced motor fluctuations and dyskinesia.

Recent review studies, however, reported that unilateral pallidotomy also has long-term effect on both cardinal symptoms (contralateral tremor and rigidity) and motor complications (contralateral dyskinesia and motor fluctuations). Currently, transcranial magnetic resonance guided focused ultrasound (MRgFUS), without necessity of opening the cranium, has been developed as a minimal invasive surgical technique, generating precise and focal thermal lesion in the brain compared to previous radiofrequency thermal lesion.

## Methods

The authors underwent world first MRgFUS pallidotomy to confirm its efficacy as well as potential side effects. We hereby demonstrated the beneficial effects of MRgFUS pallidotomy in PD patient for improving levodopa induced dyskinesia as well as motor symptoms.

